# Comparing different pneumoperitoneum (12 vs. 15 mmHg) pressures with cytokine analysis to evaluate clinical outcomes in patients undergoing robotic‐assisted laparoscopic radical cystectomy and intracorporeal robotic urinary diversion

**DOI:** 10.1002/bco2.240

**Published:** 2023-04-11

**Authors:** Nikhil Vasdev, Naomi Martin, Amon B. Hackney, John Piedad, Alexander Hampson, Gowrie‐Mohan Shan, Venkat Prasad, Michael Chilvers, Martin Ebon, Philip Smith, Gary Tegan, Karel Decaestecker, Anwar Baydoun

**Affiliations:** ^1^ Department of Urology, Lister Hospital East and North Hertfordshire NHS Trust Stevenage UK; ^2^ School of Life and Medical Sciences University of Hertfordshire Hatfield UK; ^3^ Faulty of Health and Life Sciences De Montfort University Leicester UK; ^4^ Department of Respiratory Sciences University of Leicester Leicester UK; ^5^ Department of Anaesthetics, Lister Hospital East and North Hertfordshire NHS Trust Stevenage UK; ^6^ Department of Research, Lister Hospital East and North Hertfordshire NHS Trust Stevenage UK; ^7^ Research and Development CONMED Corporation Largo Florida USA; ^8^ Department of Urology Maria Middelares General Hospital Ghent Belgium; ^9^ Department of Urology Ghent University Hospital Ghent Belgium

**Keywords:** cytokines, patient outcomes, robotic surgery, robotic urinary diversion

## Abstract

**Background:**

Robotic cystectomy is the mainstay surgical intervention for treatment‐refractory nonmuscle‐invasive and muscle‐invasive bladder cancer. However, paralytic ileus may complicate the postoperative recovery and may be a consequence of an inflammatory response associated with transient gut ischaemia. We have therefore investigated clinical, operative and inflammatory biomarker associations between paralytic ileus in the context of robotic cystectomy and intracorporeal ileal conduit urinary diversion.

**Methods:**

Prospective consective patients referred for robotic cystectomy were consented and included in the study, while patients >75 years old and converted to open procedure were excluded. The pneumoperitoneum pressure (PP) for carbon dioxide insufflation required to perform the procedure efficiently and safely was recorded (12 or 15 mmHg). We also recorded the postoperative days patients passed flatus and stools, whether they developed ileus, as well as other standard clinical and demographic data. The expression of select proinflammatory and anti‐inflammatory cytokines was determined by multiplex analysis using a cytometric bead array with changes in profiles correlated with the pressures applied and with the existence of an ileus.

**Results:**

Twenty‐seven patients were recruited, but only 20 were used in the study with 10 patients in each PP group. Seven patients were excluded all of whom had an extracorporeal ileal conduit formation. There were differences in the 40‐min shorter operative time and 1 day shorter length of stay, as well as passing flatus 1 day and stools 1.5 days earlier in the 12 mmHg compared with the 15 mmHg group. More patients had ileus in the 15 mmHg group vs 12 mmHg group (30% vs. 10.0%). These were not statistically significant. Similarly, there were no statistical differences in the expression of proinflammatory cytokines at the two different pressures or between patient groups, but there were outliers, with the median indicating nonsymmetrical distribution. By comparison, anti‐inflammatory cytokines showed some significant differences between groups, with IL‐6 and IL‐10 showing elevated levels postsurgery. No statistical difference was observed between pressures or the existence of an ileus, but the maximum levels of IL‐6 and IL‐10 detected in some patients reflect a pressure difference.

**Conclusions:**

The initial findings of this novel scientific study indicated a higher risk of paralytic ileus postrobotic cystectomy and robotic intracorporeal urinary diversion when a higher pressure of 15 mmHg is used compared with 12 mmHg. Although further studies are required to establish the linkage between cytokine profile expression, pressure and ileus, our initial data reinforces the advantages of lower pressure robotic cystectomy and intracorporeal urinary diversion in patient outcomes.

## 1 INTRODUCTION

Robot‐assisted laparoscopic procedures has revolutionised minimally invasive surgery, particularly in urological oncology. Robot‐assisted radical cystectomy (RARC) is the gold standard in treating both muscle‐invasive and high‐risk or BCG‐unresponsive nonmuscle‐invasive bladder cancer.^1^ Furthermore, advancements in intracorporeal urinary diversion techniques continue to improve operative and postoperative outcomes to benefit patients.

Ileus is a recognised postoperative complication in both robot‐assisted including standard laparoscopic surgery and open cystectomy, which impairs recovery by reducing/preventing adequate oral intake, and secondarily results in reduced mobilisation and prolonged hospital stay. It is caused by nonmechanical transient inhibition of gastrointestinal motility and results in reduced passage of flatus and stools.^2^

The pathophysiology of postoperative ileus in the context of robot‐assisted laparoscopic surgery is multifactorial. Laparoscopic surgery requires abdominal insufflation of gas and establishing a certain pneumoperitoneum pressure (PP) which can precipitate compromised mesenteric vascularity and gut ischaemia.^3^ There are studies emerging in robot‐assisted laparoscopic radical prostatectomies (RALPs), highlighting enhanced operative and postoperative recovery outcomes including reduced rates of ileus with lower PP.^4^, ^5^

Transient gut ischaemia has been associated with the development of postoperative ileus.^6^ RARCs and RALPs being performed in the steep Trendelenburg position may also potentiate this ischaemia. Furthermore, alterations in gut vascularity promote shifts in the gut barrier and microbiome, causing bacterial translocation and endotoxin release.^7^ This raises an inflammatory response, mediated by local and distant humoral factors including cytokines. Indeed, alterations in serum^8^ and peritoneal exudate cytokine levels^9^ have been associated with ileus, suggesting a relationship for further investigation. In this regard, previous studies have shown a reduction in proinflammatory cytokines and an increase in anti‐inflammatory cytokines in robot‐assisted cystectomy and abdominal surgery,^10^, ^11^ associated with a decrease in tissue damage and associated cytokine storm.^12^, ^13^ Furthermore, low‐pressure robotic surgery has been shown to improve patient outcomes and is associated with a decrease in surgical injury.^14^

With growing evidence of low‐pressure robotic surgery impacting patient outcomes positively, we carried out a study comparing PPs in RARCs and investigated whether high pressure was associated with ileus and with changes in cytokine expression with the aim of using the latter as potential biomarkers for timely management and patient recovery.

## 2 METHODS

Local and national ethical approval was sought and granted by the Health Research Authority for this study (18/SW/0250). Prospective patients aged 40–75 years referred for RARC were screened and consented for enrolment into the study. Patients converted to open procedure were excluded. Patients were randomly electronically and assigned to each PP group prospectively.

A single surgeon (NV) at a single centre performed all RARC procedures (cystoprostatectomy in males and synchronous hysterectomy/bilateral salpingo‐oophorectomy in females), a pelvic lymph node dissection and formation of an intracorporeal ileal conduit urinary diversion. All patients had an ileal conduit with a Wallace uretero‐ureteric baseplate and subsequent uretero‐ileal anastomosis. Perioperative care delivery encompassed standardised optimal anaesthetic procedures, early postoperative mobilisation and drain management was for all patients as clinically indicated. Patients were discharged to complete 28 postoperative days of low‐molecular weight heparin for venous thromboembolic prophylaxis. The operative procedure in the 25–30° Trendelenburg lithotomy position was also identical in all patients, using the da Vinci® Si robotic system in the first *N =* 15 and the da Vinci® Xi system in the following *N* = 5 (Intuitive Surgical; California, USA). The AirSeal® System (ConMed, New York, USA) insufflation system was used to maintain the chosen PP, while simultaneously facilitating filtered smoke extraction.

**FIGURE 1:**
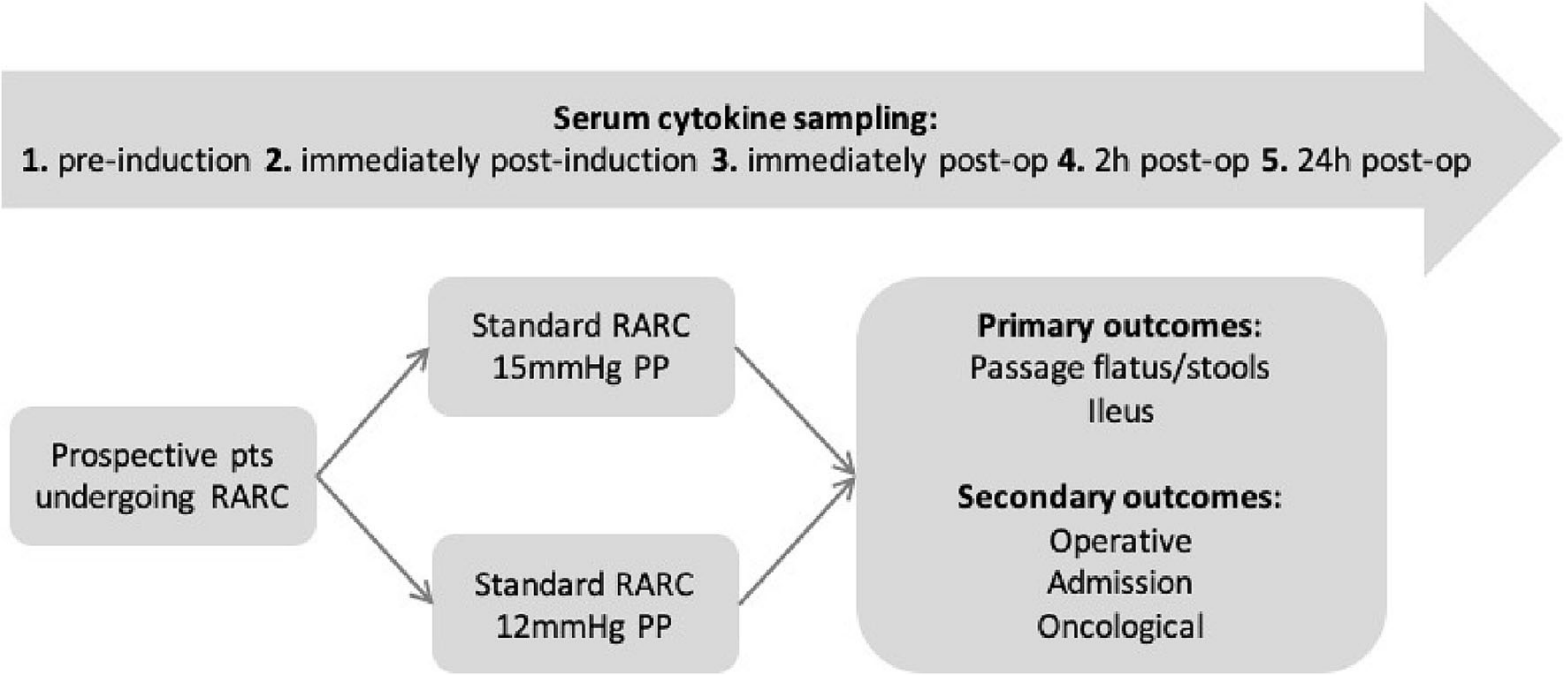
Flow diagram of project methods.

Clinical data of particular interest were the postoperative days passage of flatus and stools and presence of ileus. These data were gathered using paper and electronic medical notes. Other clinical, demographic, surgical and oncological data were also collected (Figure 1).

Venous samples were taken preanaesthesia, immediately postanaesthesia, immediately postoperatively, 2 h postoperatively and 24 h postoperatively for analysis of IL‐1β, IL‐2, IL‐4, IL‐6, IL‐10, IL‐17A, TNFα and IFNγ which represent a broad spectrum of proinflammatory and anti‐inflammatory markers. Venous blood samples were collected in EDTA vials and were kept at 2–8°C while being handled and were immediately processed. Samples underwent centrifugation in a refrigerated centrifuge at 1000 *g* at 4°C for 15 min. The plasma was aliquoted and stored at 80°C until analysis.

Cytokine analysis was carried out using the BD™ CBA Human Soluble Protein Flex Set System as described in the manufacturer's protocol. Briefly, 50 μL of sample or standard was incubated for 3 h in the dark at room temperature with an eight‐plex mixture of human soluble protein flex set capture beads. Fifty microlitres of the detection reagent, a mixture of phycoerythrin (PE)‐conjugated antibodies, was added to the samples and the mixture incubated for a further 1 h at room temperature in the dark. One millilitre of wash buffer was subsequently added to each sample and centrifuged for 5 min at 200 *g*. Supernatants were aspirated, and pellets resuspended in 350 mL of wash buffer before running the samples through an Accuri C6 flow cytometer equipped with 585, 675 and 780 nm filters. Data acquisition was set‐up according to manufacture guidelines for the Cytometric Bead Array kit for the Accuri C6. Laser configuration was set to the 2 blue 2 red mode. Gate settings were set to run with limits collecting 2400 events (300 events per plex) in the R1 gate, with the flow rate set to medium. The threshold to exclude debris for FSC‐H and SCC‐H were set at 500 000.

Data analysis was carried out using the FCAP Array v3.0.1 software. Further analysis was carried out using a one‐way analysis of variance (ANOVA) followed by a post hoc Kruskal–Wallis multiple comparisons test to determine significant differences between groups. Data are presented as median (range). Statistical significance was accepted at *P* < 0.05. Statistical analysis was performed using Prism™ software (GraphPad Prism 9.4.1.681, California, USA).

## 3 RESULTS

Twenty‐seven consecutive patients were recruited, however, *N* = 7 met the criteria for exclusion as all patients had undergone an open extracorporeal reconstruction and therefore not included in the analysis. Table 1 highlights the demographic, operative and admission data for the cohort and PP groups. Most patients were male and in the sixth decade of life, with a slight preponderance for males and increased age in the 15 mmHg group. The majority of patients were medically well, with most patients being ASA grade ‘2’, Charlson score ‘0’ and meeting the BMI cut‐off for ‘overweight’. None of these differences were statistically significant.

**TABLE 1:** Demographic and surgical parameters of the CY‐ROC trial.

Parameters	Total cohort	Pneumoperitoneum pressures			
		12 mmHg	15 mmHg	*P* *
*N*	20	10	10	–
Gender	F (30.0%)	F (40.0%)	F (20.0%)	0.628^c^
	M (70.0%)	M (60.0%)	M (80.0%)	
Age (years)^a^	65 (50–75)	63 (50–73)	69 (50–75)	0.089^b^
BMI (kg m^−2^)^a^	28 (22–34)	27 (22–31)	29.5 (23–34)	0.315^b^
Charlson score	0 (70.0%)	0 (70.0%)	0 (70.0%)	0.582^c^
	1 (20.0%)	1 (10.0%)	1 (30.0%)	
	2 (5.0%)	2 (10.0%)		
	3 (5.0%)	3 (10.0%)		
ASA grade	1 (20.0%)	1 (30.0%)	1 (10.0%)	0.582^c^
	2 (80.0%)	2 (70.0%)	2 (90.0%)	
Op time (min)^a^	240 (200–400)	240 (200–300)	280 (210–400)	0.143^b^
Blood loss (mL)^a^	50 (10–100)	50 (10–100)	30 (15–100)	0.393^b^
Blood transfusion intraoperatively or postoperatively	0	0	0	0
Passed flatus (post‐op day)^a^	2 (1–7)	2 (1–7)	3 (2–4)	0.393^b^
Passed stools (post‐op day)^a^	5.5 (4–11)	5 (4–11)	6.5 (3–10)	0.684^b^
Ileus (%)	4 (19.0%)	1 (10.0%)	3 (30%)	0.291^c^
LOS (day)^a^	9 (6–17)	8 (6–17)	9 (7–17)	0.218^b^
Readmission ≤90 days	No (90.0%)	No (80.0%)	No (80.0%)	0.709^c^
	Yes (10.0%)	Yes (20.0%)	Yes (20.0%)	
Clavien–Dindo grading	0 (90.0%)	0 (80.0%)	0 (80.0%)	0.709^c^
	2 (10.0%)	2 (20.0%)	2 (20.0%)	

Abbreviations: ASA, American Society of Anaesthesiologists; BMI, body mass index; F, female; LOS, length of stay; M, male.

^a^ Median.

^b^ Kruskal–Wallis test.

^c^ *χ* ^2^ test.

* *P* value significant at <0.05.

The oncological data for the cohort and PP groups (Table 2) show that most patients in the cohort had Ta/T1 disease, with a slightly higher proportion of T2 disease in the 12 mmHg group. These were all high‐grade transitional cell carcinomas, with more patients in the 12 mmHg group having concurrent carcinoma in situ. Half the patient cohort received intravesical BCG or mitomycin C (higher proportion in the 12 mmHg group), with 20.0% patients in each PP group receiving neoadjuvant treatment. The pathological T stage was mainly T1 for the cohort and PP groups, with small proportions of other T stages. All patients were local nodal metastatic and distant metastatic stage ‘0’. Only one positive surgical margin was found in the entire cohort (5.0%), and this patient was in the 15 mmHg group. At a mean follow‐up of 12 months, no patient has had any evidence of disease recurrence or port site metastasis.

**TABLE 2:** Oncological parameters parameters of the CY‐ROC trial.

Parameters	Total cohort	Pneumoperitoneum pressures			
		12 mmHg	15 mmHg	*P* *
Prior intravesical chemotherapy	No (50.0%)	No (40.0%)	No (60.0%)	0.656^c^
	Yes (50.0%)	Yes (60.0%)	Yes (40.0%)	
Neoadjuvant treatment	No (4.3%)	No (90.0%)	No (80.0%)	0.500^c^
	Yes (95.7%)	Yes (10.0%)	Yes (20.0%)	
cT stage	T1 (75.0%)	T1 (70.0%)	T1 (80.0%)	0.500^c^
	T2 (25.0%)	T2 (30.0%)	T2 (20.0%)	
Grade	High grade (100.0%)			–
Histology	TCC (80.0%)	TCC (60.0%)	TCC (100.0%)	0.087^c^
	TCC + CIS (20.0%)	TCC + CIS (40.0%)		
pT stage	T0 (25.0%)	T0 (20.0%)	T0 (30.0%)	0.500^c^
	T1 (50.0%)	T1 (50.0%)	T1 (50.0%)	
	T2 (5.0%)	T2 (10.0%)	T2 (0%)	
	T3 (20.0%)	T3 (20.0%)	T3 (20.0%)	
Nodal stage	0 (100.0%)			–
*N* lymph nodes^a^	9 (2–22)	9 (2–22)	10 (3–20)	0.912^b^
Metastatic stage	0 (100.0%)			–
Positive surgical margin	No (95.0%)	No (100.0%)	No (90.0%)	0.474^c^
	Yes (5.0%)		Yes (10.0%)	

^a^ Median.

^b^ Kruskal–Wallis test.

^c^ *χ* ^2^ test.

* *P* value significant at <0.05.

There was a nominally shorter length of stay (LOS) in the 12 mmHg by a median 1 day, but this was not statistically significant. Both PP groups had 10.0% of patients being readmitted within 90 postoperative days, for infective pathologies requiring only conservative pharmacological treatments (nonsignificant differences).

The 12 mmHg group had a median 40 min faster operative time, 20 mL more blood loss, 1 day quicker passage of flatus and 1.5 days quicker passage of stools when compared with the 15 mmHg group. Ileus was present in 10.0% patients in the 12 mmHg compared with 30% patients in the 15 mmHg group. These trends were not statistically significant.

**FIGURE 2:**
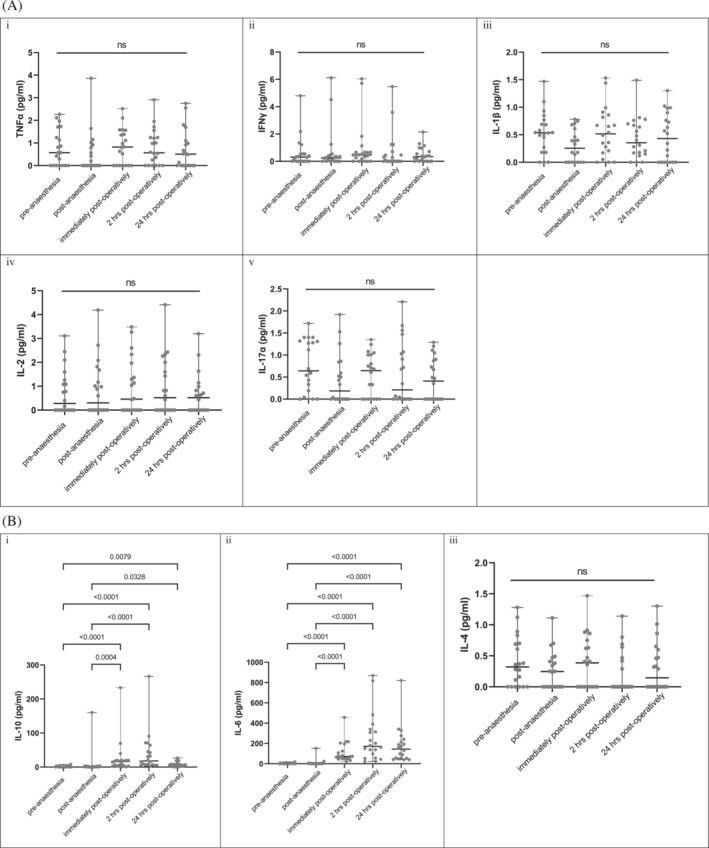
(A) Quantitative multiplex data of proinflammatory cytokine in the CY‐ROC trial. (B) Quantitative multiplex data of anti‐inflammatory cytokine in the CY‐ROC trial. Plasma cytokine levels were analysed using the BD™ CBA Human Soluble Protein Flex Set System. Samples from 20 patients under the different conditions were analysed and the data presented as median (range). Statistical analysis was performed by one‐way ANOVA, post hoc Tukey's multiple comparisons test using GraphPad Prism 9.4.1.681 and statistical significance accepted at *P* < 0.05.

Cytokine analysis show that the median values for the proinflammatory molecules TNFα, IFNγ, IL‐1β, IL‐2 and IL‐17A were not significantly altered either by pressure differences (Table 3) or between the five patient groups (Figure 2A,B). For most, the amounts detected were well within expected control levels and did not vary much in the preoperative and 24 h postoperative samples—TNFα: 0.57 (0.00–2.27) pg/mL versus 0.51 (0.00–2.75) pg/mL; IFNγ: 0.31 (0–4.80) pg/mL versus 0.35 (0.00–2.15) pg/mL; IL‐1β: 0.54 (0.00–1.47) pg/mL versus 0.43 (0.00–1.30) pg/mL; IL‐2: 0.28 (0.00–3.11) pg/mL versus 0.52 (0.00–3.20) pg/mL; IL‐17A: 0.64 (0.00–1.72) pg/mL versus 0.41 (0.00–1.29) pg/mL (Figure 2A).

**TABLE 3:** Quantitative multiplex cytokine data of the CY‐ROC trial comparing low versus high pneumoperitoneum pressure.

Cytokine (pg/mL)		Preanaesthesia		Immediately postanaesthesia		Immediately postoperatively		2 h postoperatively		24 h postoperatively	
	Pressure (mmHg)	12	15	12	15	12	15	12	15	12	15
IL‐1β	Median	0.49	0.58	0.39	0.00	0.54	0.35	0.21	0.40	0.57	0.34
	Minimum	0.00	0.00	0.00	0.00	0.00	0.00	0.00	0.00	0.00	0.00
	Maximum	1.47	1.10	0.75	0.78	1.44	1.53	0.78	1.49	1.02	1.30
IL‐2	Median	0.00	1.08	0.60	0.00	0.00	1.04	0.00	0.82	0.60	0.44
	Minimum	0.00	0.00	0.00	0.00	0.00	0.00	0.00	0.00	0.00	0.00
	Maximum	2.10	3.11	2.08	4.19	3.48	3.27	2.43	4.41	3.20	2.31
IL‐4	Median	0.27	0.38	0.25	0.00	0.36	0.41	0.00	0.00	0.00	0.33
	Minimum	0.00	0.00	0.00	0.00	0.00	0.00	0.00	0.00	0.00	0.00
	Maximum	0.89	1.28	0.67	1.11	0.91	1.47	0.80	1.14	1.30	0.86
IL‐6	Median	1.48	2.24	1.82	1.21	73.04	52.36	180.80	161.40	130.60	171.10
	Minimum	0.00	0.00	0.00	0.00	23.14	33.59	21.83	43.58	40.48	39.27
	Maximum	4.44	18.67	7.49	151.80	218.20	456.70	868.90	818.10	820.20	341.60
IL‐10	Median	0.49	1.69	1.07	1.13	17.67	9.32	15.91	20.45	6.79	7.64
	Minimum	0.00	0.00	0.00	0.00	0.18	2.15	2.60	4.96	0.00	0.00
	Maximum	2.85	7.98	2.58	160.40	233.50	20.11	266.70	71.36	19.11	26.63
IL‐17A	Median	0.43	1.32	0.04	0.50	0.68	0.33	0.07	0.35	0.47	0.35
	Minimum	0.00	0.00	0.00	0.00	0.00	0.00	0.00	0.00	0.00	0.00
	Maximum	1.27	1.72	1.53	1.92	1.35	1.24	2.21	1.56	1.29	1.05
TNFα	Median	0.81	0.57	0.00	0.00	1.11	0.63	0.36	0.59	0.54	0.00
	Minimum	0.00	0.00	0.00	0.00	0.00	0.00	0.00	0.00	0.00	0.00
	Maximum	2.11	2.27	1.64	3.86	2.52	1.58	2.91	1.55	2.75	1.80
IFNγ	Median	0.00	0.35	0.08	0.27	0.46	0.52	0.00	0.31	0.20	0.40
	Minimum	0.00	0.00	0.00	0.00	0.00	0.00	0.00	0.00	0.00	0.00
	Maximum	1.22	4.80	1.24	6.11	1.13	6.04	1.23	5.47	1.15	2.15

*Note*: Plasma cytokine levels were analysed using the BD™ CBA Human Soluble Protein Flex Set System. Medians were determined from 11 samples in the 12 mmHg and nine in the 15 mmHg groups, respectively.

By comparison, the IL‐10 and IL‐6, which are considered to be anti‐inflammatory under certain conditions, increased significantly in the immediate and 2 h postoperative samples (presurgery vs. 2 h postoperative—IL‐6: 1.48 [0.00–18.67] pg/mL vs. 171.10 [21.83–868.90] pg/mL; IL‐10: 0.78 [0.00–7.98] pg/mL vs. 18.18 [2.60–266.70] pg/mL) (both *P* < 0.0001; Figure 2Bi and ii). Both cytokines remained partially elevated 24 h postsurgery. IL‐4 was detected at low levels and did not change significantly between groups or pressures (Figure 2Biii and Table 3).

## 4 DISCUSSION AND CONCLUSIONS

Our data highlight interesting trends in perioperative outcomes with lower PP in RARC and add to the growing body of evidence favouring lower PP robotic, as well as laparoscopic surgery in general. We highlight faster passage of flatus and stools and reduced risk of ileus in 12 versus 15 mmHg. The lower PP was also associated with shorter LOS, more blood loss and quicker operative time. Interestingly, postoperative complication rates and resultant admission were not different between the groups. While not statistically significant, these trends which corroborate other findings from other studies are promising and may relate to the smaller study sample size.

A US unit retrospectively reviewed records of *N* = 200 consecutive RALPs to investigate operative parameters and postoperative outcomes between 12 and 15 mmHg PPs (1:1 subject ratio).^15^ They found that 12 mmHg was also associated with less ileus (4% vs. 8%, statistically nonsignificant). Additionally, operative times were slightly quicker with lower pressures; and complication and readmission rates were similar across PPs. Retrospective data from the same unit supported these findings, with more patient numbers.^16^ In particular, ileus was present in 10% 12 mmHg versus 25% 15 mmHg (*P* = 0.014), and LOS was 1.49 versus 1.76 days, respectively (*P* = 0.022). A similarly randomised and double‐blinded prospective trial from this group compared 12 to 8 mmHg.^5^ Ileus was present in 2.0% in the 8 mmHg group versus 4.8% with 12 mmHg (*P* = 0.45). The higher pressure group had a 0.2 day longer LOS (*P* < 0.05).

Another US study looking again at consecutive RALPs, but this time at an even lower pressure of 6 (*N* = 300) versus 15 mmHg (*N* = 300) show significant promise.^4^ The lower pressure group was also associated with 20 mL more blood loss, shorter LOS (0.5 vs. 1 days) and same day discharges (43.3% vs. 0, all *P* < 0.001). Only one patient had ileus in their cohort (15 mmHg). Patients had statistically better postoperative pain control in the 6 mmHg group. However, contrary to our data, the 15 mmHg had higher postoperative rates of overall complications (8.7% vs. 4.0%, *P* = 0.02), number of 30‐day emergency attendances (11.0% vs. 5.0%, *P* < 0.01) and admissions (5.7% vs. 1.0%, *P* < 0.01).

A very recent French Phase 3 randomised controlled trial on laparoscopic colectomies (*N* = 127) has also highlighted favourable outcomes in terms of postoperative pain and LOS in the 7 mmHg (*N* = 62) compared with the 12 mmHg group.^17^ This study also found lower rates of ileus in the low‐pressure group (3% vs. 11%, *P* = 0.041), with no impairments to surgical safety or quality.

Mastroianni et al.^18^, ^19^ have shown the benefit a reduction in blood transfusion rates in patients undergoing RARC when compared with open cystectomy, confirming a significant benefit in favour of RARC with intracorporeal. Our data confirm that none of our 20 patients had any intraoperative or postoperative blood transfusion. Presicce et al.^20^ evaluated the safety of RARC and intracorporeal urinary diversion with late complications in patients who underwent RARC with intracorporeal urinary diversion (ICUD). Results are encouraging and in line with findings from a historical series of open radical cystectomy (ORC). Catto et al.^21^ in the iROC clinical trial RARC with intracorporeal urinary diversion versus ORC resulted in a statistically significant increase in days alive and out of the hospital over 90 days. However, the clinical importance of these findings remains uncertain.

Our unit carried out a similar study investigating operative, clinic‐demographic and oncological differences between PPs in robotic prostatectomies in *N* = 10 patients. We also found that lower pressures were associated with reduced rates of ileus, and furthermore, this was associated with alterations in postoperative cytokine levels favouring an anti‐inflammatory response.^22^ The latter has not been explicitly confirmed in the current study, with the medians for IL‐1β, IL‐2, IL‐17A, TNFα and IFNγ showing no statistically significant differences between pressures or study groups.

Unlike the proinflammatory cytokines, IL‐6 and IL‐10 were expressed at low levels presurgery but increased significantly immediately postsurgery, peaking 2 h after and remained partially elevated over 24 h. As with the proinflammatory cytokines, we have not demonstrated a statistical difference between levels at 12 and 15 mmHg pressures which may be due to large variabilities in the expression of cytokines amongst individuals within cohorts and to the small numbers of participants in each group. This therefore warrants a larger study that is powered to ensure statistical differences can be clearly demonstrated. We acknowledge that in our pilot study, we have not looked into pain score and will be in future planned trials. We acknowledge the limitations in our study including limited numbers of 20 patients only but a larger multicentre is currently in preparation where the impact of different PPs during RARC and intracorporeal robotic urinary diversion. Oncological follow‐up is currently at 12 months, reassuringly no disease recurrence has been identified but longer data collection will continue.

Low‐pressure robotic surgery shows promising positive trends in operative and postoperative recovery parameters.^23^ At the time of writing, we are the only study to look at ileus across different PPs in RARC. The similar findings of lower rates of ileus in lower versus higher PP are interesting, despite an important caveat that RARCs involve significant bowel handling and dissection compared with RALPs. This suggests that PP choice is an important shared predictor. While a growing body of evidence has emerged for RALPs and other laparoscopic procedures in general, there need to be more research with RARCs, and this pilot project has built a foundation for a larger randomised trial. Consideration of other potential confounding operative factors such as extent of and length of time during Trendelenburg position could elucidate technical parameters for optimisation and highlight pathophysiological mechanisms. Furthermore, looking at how cytokine levels associate with these findings will also be clinically relevant.

## AUTHOR CONTRIBUTIONS

*Data collection and processing*: Nikhil Vasdev, John Piedad, Alexander Hampson, Martin Ebon, Anwar Baydoun, Naomi Martin, Amon B. Hackney. *Operative and clinical input*: Nikhil Vasdev, John Piedad, Alexander Hampson, Michael Chilvers, Gowrie‐Mohan Shan and Venkat Prasad. *Manuscript preparation*: John Piedad, Karel Decaestecker, Gary Tegan, Anwar Baydoun and Nikhil Vasdev. *CI and overall project supervision*: Nikhil Vasdev.

## CONFLICT OF INTEREST STATEMENT

The authors declare no conflicts of interest.
